# Impact of Germination on the Edible Quality and Nutritional Properties of Brown Rice Noodles

**DOI:** 10.3390/foods13132152

**Published:** 2024-07-08

**Authors:** Ruiyun Chen, Xudong Yan, Mingxi Cai, Jiamei Cai, Taotao Dai, Yunfei Liu, Jianyong Wu

**Affiliations:** 1 Jiangxi General Institute of Testing and Certification, Nanchang 330052, China; 2 State Key Laboratory of Food Science and Resources, Nanchang University, Nanchang 330047, China; 3 Institute of Applied Chemistry, Jiangxi Academy of Sciences, Nanchang 330096, China

**Keywords:** brown rice, germination, noodles, edible quality, nutrition

## Abstract

Brown rice noodles are increasingly favored by consumers for their health benefits; however, their development is hindered by their poor edible qualities. The effect of germination on the cooking, textural, organoleptic and nutritional qualities of brown rice pasta was investigated. In comparison to ungerminated brown rice noodles, germination resulted in a shorter cooking time, reduced cooking losses, and decreased hardness and adhesion of noodles as well as reduced bitter taste. These changes can be attributed to germination altering the basic composition of brown rice. Meanwhile, the contents of γ-aminobutyric acid, free phenolic acid, and bound phenolic acid increased by 53.43%, 21.71%, and 7.14%, respectively, while the content of resistant starch de-creased by 21.55%. Sprouting is a promising strategy for improving the edible quality and nutritional properties of brown rice noodles.

## 1. Introduction

Rice noodles are one of the staple foods in Southern China and Southeast Asia. They are made from their main ingredients, such as polished rice and brown rice, through a series of processing steps including washing, soaking, crushing or grinding, fermenting or not fermenting, gelatinizing, extruding or cutting, and drying or not drying; this results in products that are either fine and filamentous or wide and flat in shape [1]. In recent years, consumers have increasingly valued health alongside taste. Brown rice and its derivative products (such as brown rice noodles) have also become favored by consumers. Compared to polished rice, brown rice contains higher levels of non-starch nutrients such as protein, minerals, fatty acids, dietary fiber, and phenolic compounds, as these nutrients are concentrated in the bran layer. As well as providing daily energy, increased consumption of brown rice can also help reduce the incidence of chronic diseases [2]. However, rice noodles processed from polished rice are known for their springy and smooth texture, whereas brown rice, containing a significant amount of bran fiber, not only affects the processing of rice noodles but also leads to poorer quality upon cooking. Sun et al. [3] significantly enhanced the quality of whole grain rice noodles by incorporating extruded rice bran into refined white rice. Yi et al. [4] demonstrated that rice bran fermentation effectively improves the texture, enriches the flavor, and enhances the antioxidant activity of brown rice noodles. Geng et al. [5] found that the eating quality of brown rice noodles was improved when brown rice was treated with ultrasound-assisted cellulase. These methods essentially involve physical or chemical modification of the bran layer fibers, reducing their impact on the edible and nutritional quality of brown rice noodles.

Germination, as a low-cost and high-benefit technology, has been applied in the processing of grains [6]. Germination activates endogenous enzymes in brown rice, which alters the structure and functionality of components like starch, protein, and dietary fiber. This process potentially enhances the cooking and edible quality of brown rice noodles [7]. Furthermore, germination can also promote a variety of bioactive substances like polyphenols and γ-aminobutyric acid (GABA), further enhancing the nutritional value of the noodles. Among them, GABA is an important inhibitory neurotransmitter, with anti-anxiety, sleep improvement, and blood-pressure-lowering effects. However, the content of GABA in brown rice is very low, usually existing in the form of small-molecule compounds. Germination can promote the decarboxylation of glutamic acid to produce GABA [8,9,10]. We hypothesized that germination would improve the eating quality and nutritional value of brown rice noodles.

Therefore, this study aimed to investigate the impact of germination on the quality of brown rice noodles, with a particular focus on their eating quality and nutritional value. Edible qualities including appearance, microstructure, cooking quality, textural characteristics, taste, and flavor were analyzed. In addition, the nutritional properties of brown rice noodles include gamma-aminobutyric acid, polyphenol content, and the in vitro digestive properties of starch. The aim of the present study is to provide a theoretical basis for the future development of nutritious and tasty whole grain-based foods.

## 2. Materials and Methods

### 2.1. Materials

Brown rice (younian) was purchased from a local supermarket. A total starch content assay kit was obtained from Megazyme International Ireland Ltd. (Bray, Ireland). Bromophenol blue-methyl red indicator and furfural were sourced from Solabo Biological Technology Co., Ltd. (Beijing, China). Gallic acid (chromatographically pure) and methanol (chromatographically pure) were procured from West Long Science Co., Ltd. (Shantou, China). γ-Aminobutyric acid standard, pancreatin, monosaccharide standard set, and phenolic acid standard set were acquired from Sigma Aldrich (Shanghai, China). All other reagents were at least analytical-grade reagents.

### 2.2. Preparation of Germinated Brown Rice

Germinated brown rice was prepared according to the method previously reported by Caceres et al. [11]. The brown rice was washed with distilled water, surface-sterilized with NaClO (0.1%) for 30 min, and then washed with distilled water until the pH value was neutral. Subsequently, the drained brown rice was transferred to plastic plates and covered with wet germination paper after soaking in deionized water at 28 °C for 12 h. The plastic plate was then placed in an incubator set to 28 °C with 90% humidity for 18 h to facilitate germination.

### 2.3. Preparation of Germinated Brown Rice Noodles

The germinated brown rice was pulverized with a colloid mill (BT-JTM-01, Wenzhou Botai Machinery Technology Co., Ltd., Wenzhou, China), and then the most moisture was removed by centrifugation and loosened (passed through a 40-mesh sieve). The germinated brown rice flour was transferred to an oven set at 45 °C until its moisture content reached approximately 35%. The germinated brown rice flour was then fed into the feeding device of a twin-screw extruder (FMHE36-24, FUMACH, Changsha, China), using a die with a diameter of 2.2 mm. The extrusion parameters were set as follows: the feed rate was 22.5 kg/h, screw speed was 100 rpm, and the temperature of the five sections of the twin-screw extruder was set at 105 °C. The extruded noodles were regenerated at 40 °C and 80% humidity for 8 h. Finally, they were air-dried at room temperature and sealed in self-sealing bags. The moisture contents of BRN and GBRN were 10.61% and 10.77%, respectively. The brown rice noodles and germinated brown rice noodles were named BRN and GBRN, respectively.

### 2.4. Determination of Cooking Quality

Optimal cooking time: The noodles (15 g), approximately 10 cm in length, were heated in 600 mL of boiling distilled water. As the noodles neared completion, segments were extracted every 15 s and delicately compressed between two transparent glass plates. The cooking process was deemed finished upon the disappearance of the white core, and the corresponding time was noted [12].

Cooking loss: Noodles (5.0 g) of about 5 cm long were added to 200 mL of boiling distilled water and heated for the optimum steaming time. After cooking, the noodles were placed on a filter, rinsed with 50 mL of distilled water for 30 s, and drained for 5 min. Then, we placed the noodles in a 105 °C drying oven and dried to constant weight (m). The cooking loss was calculated using the following equation [13]:

Cooking loss (%) = (M − m)/M × 100(1)

where M is the constant weight (g) of 5 g dry noodles measured at 105 °C, and m is the constant weight (g) of 5 g dry noodles after cooking and measured at 105 °C.

Breakage rate: Twenty rice noodles, each 25 cm in length, were heated in distilled water at 100 °C for the optimal cooking time, and the number of broken noodles (n) was then recorded. The breakage rate was calculated using the following equation [13]:

Breakage rate (%) = n/20 × 100(2)

where n is the number of broken rice noodles after cooking 20 noodles.

### 2.5. Determination of Texture Properties

The determination of the texture characteristics of germinated brown rice noodles followed the method outlined by Luo et al. [14]. Three strands of cooked germinated brown rice noodles were chosen for texture profile analysis (TPA) using a texture analyzer (XT-plus, Stable Micro Systems, Surrey, UK) under specified conditions: using a P36/R probe, with an applied force of 5 g, a compression ratio of 90%, and with pre-test, and post-test speeds of 1 mm/s, 2 mm/s, and 2 mm/s, respectively.

### 2.6. Appearance and Microscopic Structure of Germinated Brown Rice Noodles

Appearance: In order to better illustrate the color differences of the noodles, a spectrophotometer (CM-23d, Konica Corporation, Tokyo, Japan) was employed to determine the colors of raw and cooked noodles [15]. In this context, L* = 0 denotes black, while L* = 100 signifies white; a* values with a positive sign indicate a reddish hue, whereas negative values denote a greenish hue, with larger magnitudes indicating more pronounced deviations; b* values with a positive sign suggest a yellowish tint, while negative values indicate a bluish tint. The whiteness index (WI) was calculated according to a previous report [2].

(3) $$WI=100-\sqrt{{\left(100-L*\right)}^{2}+{\left(a*\right)}^{2}+{\left(b*\right)}^{2}}$$

Microscopic structure: Noodles cooked for the optimal steaming time were freeze-dried, and pressure was applied along the transverse axis of the noodles using a blade to induce natural fracturing. The fractured surface of the noodles was positioned facing upward, fixed with conductive adhesive, and then coated with a layer of metal film on the cross-section. The microscopic structure of the noodles was observed using a scanning electron microscope (Regulus 8100, HITACHI, Tokyo, Japan) at an acceleration voltage of 20 kV, with magnifications of 40, 200, and 500 times, respectively.

### 2.7. Odor and Flavor of Germinated Brown Rice Noodles

Electronic nose: Cooked noodles (5 g) and saturated NaCl solution (50 mL) were introduced into a 50 mL headspace vial. Testing was conducted using an electronic nose (PEN3, Airsense Analytics GmbH, Schwerin, Germany) after equilibration at 25 °C for 30 min. The testing parameters were as follows: sample preparation for 5 s, sampling interval of 1 s, automatic sensor cleaning for 120 s, sensor zeroing for 5 s, sample injection flow rate of 600 mL/min, and a testing duration of 60 s, repeated thrice [16].

Electronic tongue: Dried noodles (3.5 g) were cooked and vortexed at room temperature for 5 min after adding deionized water, followed by centrifugation at 6000× *g* for 10 min to obtain the supernatant. The supernatant was diluted to 100 mL and then poured into the beaker of an automatic sample injector for the electronic tongue (SA402B Plus, Insent Inc., Tokyo, Japan). The parameters for the electronic tongue instrument were set as follows: a sample collection time of 120 s, stirring rate of 60 rpm, sampling interval of 1 s, and a taste sensor cleaning time of 20 s, with each sample measured in triplicate [17].

### 2.8. Basic Components of Germinated Brown Rice Noodles

The total starch content was determined according to the protocol specified in the total starch assay kit (Megazyme International Ireland Ltd., Bray, Ireland). The lipids, protein, and dietary fiber contents were determined using AACC-approved methods 30-26, 46-08, and 32-05, respectively (AACC, 2010).

### 2.9. Determination of γ-Aminobutyric Acid in Brown Rice Noodles

Brown rice noodles (1 g) and deionized water (10 mL) were introduced into a 50 mL centrifuge tube. The mixture was blended in a magnetic stirrer for 2 h at 30 °C followed by centrifugation at 5000× *g* for 10 min. The supernatant was carefully collected, and the residue underwent two additional reprocessing steps. The combined filtrates were concentrated to 5 mL at 45 °C under reduced pressure. Next, the filtrate (1.5 mL) was transferred into a 10 mL colorimetric tube. Sequentially, 0.6 mL of 0.2 M sodium tetraborate buffer solution (pH 9.0), 3.0 mL of 6% resorcinol solution, and 1.2 mL of 6.5% sodium hypochlorite solution were added. The mixture was thoroughly blended and then incubated in a boiling water bath for 10 min, followed by cooling in an ice bath for 20 min with continuous shaking. Once the solution achieved a blue-green color, the ethanol solution (6.0 mL, 60 *w*/*w*) was added. After thorough agitation, the mixture stood for 40 min. Finally, the absorbance value was determined under the wavelength of 645 nm [18].

### 2.10. Polyphenols in Germinated Brown Rice Noodles

#### 2.10.1. Extraction of Free Polyphenols

The noodles (1.0 g) were added to 30 mL of acidified ethanol (prepared by mixing 95% ethanol and 1 M HCl in an 85:15 ratio, *v*/*v*) at 4 °C. The mixture was stirred magnetically for 15 min, followed by centrifugation at 2160× *g* for 5 min to obtain the supernatant. The residue underwent extraction using the same procedure three times, with all the supernatants being combined. The organic solvent was evaporated at 45 °C, and the residue was dissolved in 5 mL of 50% chromatographic-grade methanol. Finally, the extracts were stored at −20 °C until further use [2].

#### 2.10.2. Extraction of Bound Polyphenols

The residual organic reagents in the residue remaining after free phenol extraction were removed using a nitrogen evaporator. After adding 20 mL of n-hexane and mixing thoroughly, the mixture was magnetically stirred for 10 min, followed by centrifugation at 2160× *g* for 5 min to discard the supernatant. The n-hexane was then evaporated using a nitrogen evaporator. Next, NaOH solution (20 mL, 2 M) was added and allowed to dissolve for 1 h in a nitrogen stream. The pH was subsequently adjusted to 2.0 using concentrated hydrochloric acid. Following this, ethyl acetate (20 mL) was added and stirred for 2 min, followed by centrifugation at 2160× *g* for 10 min to obtain the supernatant. The extraction was repeated 4 times, and the extracts were combined. Organic reagents were removed from the combined solution using rotary evaporation at 45 °C, and the residue was subsequently dissolved in 5 mL of 50% chromatography-grade methanol. The solution was stored at −20 °C until further use [19].

#### 2.10.3. Determination of Total Phenolic Content

The distilled water (1 mL) and polyphenol extract (250 μL) were added a 15 mL centrifuge tube. Subsequently, Folin–Ciocalteu reagent (0.25 mL) was introduced and reacted for 6 min. Then, Na_2_CO_3_ solution (2.5 mL, 7%) and distilled water (6 mL) were sequentially added and thoroughly mixed. The mixture was then reacted for 90 min away from light, and the absorbance values were measured at a wavelength of 760 nm. The total polyphenol content was expressed as micrograms of gallic acid equivalent per gram of dry weight (µg GAE/g DW) [19].

### 2.11. Resistant Starch Content

To simulate the particle size of noodles after chewing, cooked noodles were freeze-dried and pulverized to 20–30 mesh particles. The RS content of the samples was determined using a previously reported method with minor modifications [20]. Sample (200 mg) was placed in a 50 mL centrifuge tube with a stirrer, followed by the addition of 20 mL of sodium acetate buffer solution (0.2 M, pH 5.2). The mixture was then magnetically stirred at 100 rpm for 10 min at 37 °C. Subsequently, 200 μL of mixed enzyme solution (396 U/mL amyloglucosidase and 360 U/mL α-amylase) was added and incubated in a magnetic stirring water bath at 37 °C and 100 rpm. A small amount of the enzymatic solution was taken at 20 min and 120 min, and the glucose content was determined using a Megazyme kit (Megazyme International Ireland Ltd., Bray, Ireland). Resistant starch content was calculated according to the following formula:

(4) $$RDS\left(\%\right)=\frac{\left(G_{20}-G_{0}\right)\times 0.9}{TS}\times 100$$

(5) $$SDS\left(\%\right)=\frac{\left(G_{120}-G_{20}\right)\times 0.9}{TS}\times 100$$

(6) $$RS\left(\%\right)=\frac{TS-\left(RDS+SDS\right)}{TS}\times 100$$

where RDS, SDS and RS indicate rapidly digested starch, slowly digested starch and resistant starch, respectively; G0, G20, and G120 are the glucose content produced at 0, 20, and 120 min of enzymatic digestion, respectively; TS is the total starch mass of the noodles; and 0.9 is the conversion factor of the starch.

### 2.12. Statistics Analysis

All tests were repeated at least three times, and the experimental results are presented as mean ± standard deviation. Statistical significance analysis of the experimental data was conducted using SPSS 25.0 software through an independent-samples *t*-test, with the significance level set at *p* < 0.05. Graphs were prepared using Origin 2018 software.

## 3. Results and Analysis

### 3.1. Effect of Germination on the Cooking, Textural, and Sensory Quality of Brown Rice Noodles

#### 3.1.1. Cooking and Textural Properties

High-quality rice noodles should exhibit a short cooking time, low cooking loss, and minimal breakage rate [21]. As shown in Table 1, the optimal cooking time and cooking loss for BRN were 709.67 s and 10.07%, respectively. Following germination, the optimal cooking time and cooking loss of GBRN significantly decreased to 667.33 s and 8.30%, respectively, while the breakage rate remained statistically unchanged (*p* < 0.05). Cooking losses reflect the ability of the noodles to maintain their intact structure during the cooking process [22]. The observed reduction in cooking loss suggests a diminished disruption of the gel network structure by boiling water, potentially due to the increased soluble dietary fiber content in brown rice during germination. Soluble dietary fiber supports the formation of a robust starch–protein network during noodle processing, thereby enhancing the gel strength of the noodles [23].

High-quality noodles are characterized by appropriate hardness, adhesiveness, and chewiness [24]. According to Table 1, BRN exhibited hardness, adhesiveness, and chewiness values of 493.71 g, 1.25 g·s, and 624.88 g, respectively. Following germination, these attributes significantly decreased (*p* < 0.05) to 438.15 g, 0.82 g·s, and 473.46 g, respectively. The hardness of rice noodles correlates positively with amylose and protein content, while chewiness depends on both hardness and adhesiveness [24,25]. Hence, the observed reduction in hardness and chewiness in germinated brown rice noodles is likely attributable to the activation of endogenous enzymes in brown rice during germination, resulting in the degradation of amylose and proteins [26]. The reduction in adhesiveness can be attributed to the decrease in cooking losses. Lower cooking losses result in fewer starch gel fragments and less leached starch adhering to the surface of the rice noodles, thereby reducing their adhesiveness [27].

#### 3.1.2. Appearance and Color

**Figure 1:**
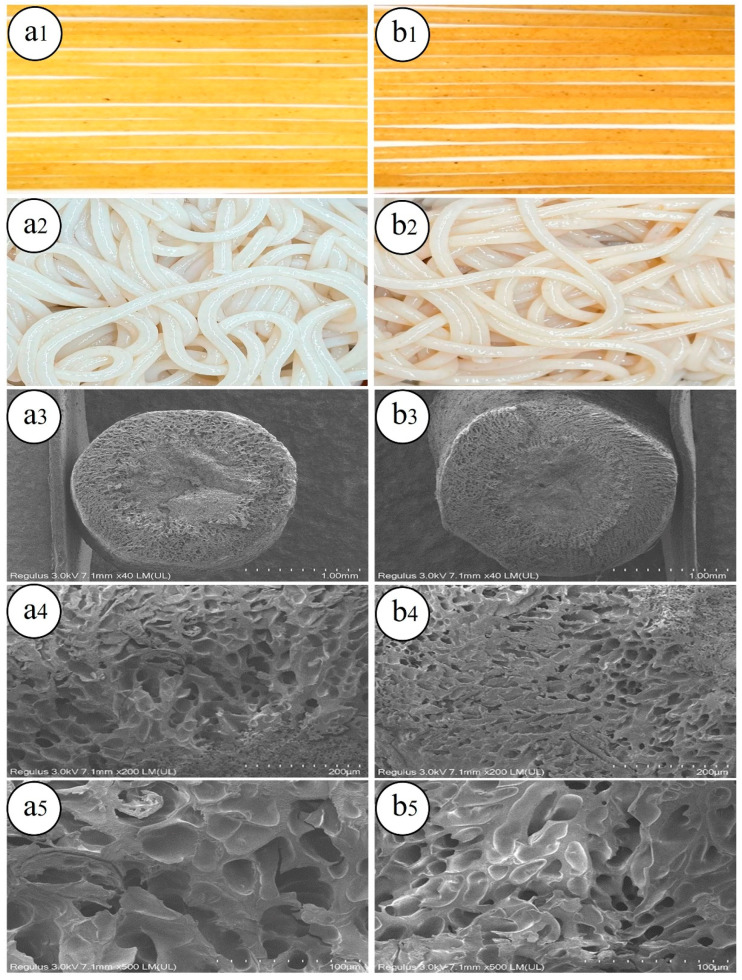
Photographs and microstructure of brown rice noodles. (**a1**) Uncooked brown rice noodles; (**b1**) uncooked germinated brown rice noodles; (**a2**) cooked brown rice noodles; (**b2**) cooked germinated brown rice noodles. The microstructures of cooked brown rice noodles in (**a3**–**a5**) and (**b3**–**b5**) are the microstructures of BRN and GBRN magnified 40, 200, and 500 times, respectively. BRN: brown rice noodles, GBRN: germinated brown rice noodles.

The appearance and color of brown rice noodles are critical factors influencing consumer acceptance [28]. As depicted in Figure 1, both types of brown rice noodles exhibited uniform thickness and smooth surfaces, with GBRN displaying a darker color compared to BRN both before and after cooking. The whiteness index (WI) corroborates this observation, representing the overall whiteness of the noodles (Table 2). Following germination, the a* and b* values of brown rice noodles significantly increased (*p* < 0.05), while the L* and WI values showed no significant change. After cooking, there was a significant decrease in L*, b*, and WI values, coupled with a significant increase in the a* value (*p* < 0.05). These findings suggest that germination darkened the color of brown rice vermicelli both before and after cooking. This color alteration may stem from the hydrolysis of starch and proteins into reducing sugars and amino acids during germination, which subsequently undergo the Maillard reaction under high temperatures and pressures, leading to the browning of the noodles. Similarly, a decreasing trend in a* and b* values was observed in the preparation of noodles from germinated wheat compared to ungerminated wheat [29].

#### 3.1.3. Microstructure

The microstructure of BRN and GBRN under an electron microscope is shown in Figure 1. The honeycomb-like porous structure of the brown rice noodles’ cross-sections is clearly shown; this structure is due to moisture sublimation. Compared to BRN (Figure 1(a4,a5)), GBRN (Figure 1(b4,b5)) had smaller, more uniform, and denser pores. This structure helps to reduce the leaching out of starch molecules during cooking and improves the hardness of BRN. This phenomenon may arise from the action of endogenous enzymes during the germination process, which disrupts the structure of rice bran. This disruption reduces the particle size of the bran, rendering it more susceptible to encapsulation by starch. Consequently, a stronger three-dimensional gel network is formed [5]. Similarly, gel prepared from sprouted red quinoa exhibits denser pores and smaller pore sizes [30].

#### 3.1.4. Smell and Taste

**Figure 2:**
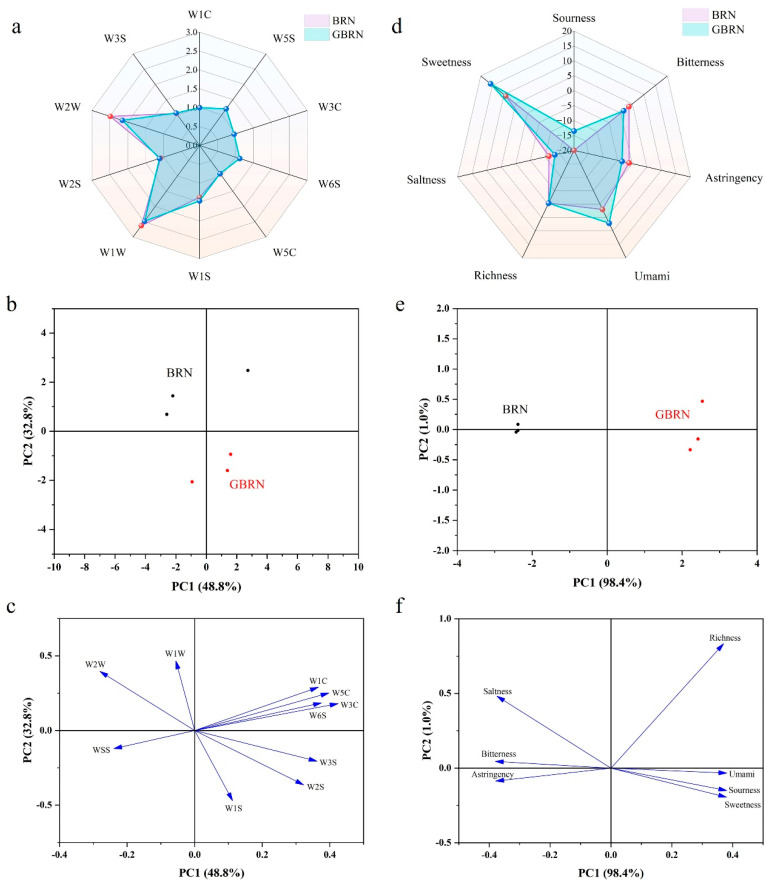
Electronic nose (**a**) and electronic tongue (**d**) radar maps of rice noodles. Score (**b**) and loadings’ plots (**c**) for the principal component analysis of the GBN. Score (**e**) and loadings’ plots (**f**) for the principal component analysis of the GBN. BRN: brown rice noodles, GBRN: germinated brown rice noodles.

An electronic nose is a system that uses gas sensors to identify odors; it is capable of continuously detecting odor conditions in real time at a specific location over a period of time. In this experiment, ten types of sensors with varying sensitivities to different gases were used. The response values from each sensor were compiled to create a radar chart, as shown in Figure 2a. The flavor radar plots showed that the flavors of the samples nearly overlap, with higher responses in the W1W and W2W sensors, which were sensitive to sulfides as well as ketones and aldehydes, respectively. To further determine the differences in the flavor profiles of each sample, principal component analysis (PCA) was conducted (Figure 2b,c). Figure 2c presents the score plots of brown rice noodles, which clearly respond to differences between the samples. The total variance contribution of PC1 (48.8%) and PC2 (32.8%) was 81.6%, indicating that these two principal components effectively reflected the differences in the flavor characteristics of brown rice noodles. Brown rice noodles exhibit a greater distinction on PC2, with BRN located in the positive quadrant and GBRN in the negative quadrant of PC2. Loading plots derived from electronic nose responses can effectively assess the individual contribution of each sensor in characterizing brown rice noodles (Figure 2c). BRN showed elevated responses in the W1W and W2W sensors, suggesting higher concentrations of sulfides as well as ketones and aldehydes. Excessive amounts of sulfur-containing compounds can generate pungent odors and decrease consumer acceptability [31]. Conversely, GBRN exhibited heightened responses in the W1S and W2S sensors, indicating higher levels of methane and alcohol.

An electronic tongue is an artificial intelligence taste recognition technology used to identify the overall taste characteristics of a sample. It comprises seven sensors, including sensors for sourness, saltiness, umami, sweetness, and bitterness, as well as two general sensors for richness and astringency. The response values of these sensors indicate their sensitivity to the respective tastes [17]. The response values from each sensor were used to create a radar chart, as shown in Figure 2b. As illustrated in Figure 2b, brown rice noodles had large response values for the sweetness, bitterness, and umami sensors. The variability of the samples was further analyzed using PCA (Figure 2e,f). As illustrated in Figure 2e, the first two principal components explain 99.4% of the total variance. BRN and GBRN exhibited significant differences along PC1. Specifically, BRN is located in the negative quadrant of PC1, while GBRN is positioned in the positive quadrant of PC1. Based on the loadings’ plots (Figure 2f), BRN was characterized by more bitter and astringent flavours, whereas GBRN was sweeter, more acidic, and umami. Germination reduced the bitterness value of brown rice noodles, probably because germination breaks down some of the starches and proteins in brown rice into soluble sugars and sweet amino acids, which neutralize the bitterness of the noodles [32]. Additionally, germination may degrade some bitter-tasting amino acids, further reducing the bitterness of the brown rice noodles [33]. A similar phenomenon was observed by Luo et al. [34]. Cakes prepared using sprouted brown rice were sweeter and less astringent compared to those made with regular brown rice. Overall, germination enhanced the flavor and taste of the brown rice noodles.

### 3.2. Effect of Germination on the Nutritional Quality of Brown Rice Noodles

The approximate composition of the brown rice noodles is shown in Table 3. The total starch, dietary fiber, lipid, and protein contents of brown rice noodles (BRN) were 81.79%, 3.56%, 0.65%, and 6.90%, respectively. After germination, the total starch content in the noodles significantly decreased to 75.38%, while the dietary fiber and lipid contents significantly increased to 6.15% and 0.73%, respectively. The protein content remained unchanged (*p* < 0.05). The decrease in starch content may be due to the degradation of starch by amylase enzymes during germination to produce other substances of lower molecular weight, such as dextrins [35]. There was a significant increase in total dietary fiber, likely attributable to the formation of new cell walls during germination [36]. Similarly, Ukpong et al. [37] found that germination reduced the total starch content and dietary fiber content in brown rice.

γ-Aminobutyric acid (GABA) is an important inhibitory neurotransmitter that benefits human health [8]. Table 3 indicates that the GABA contents in BRN and GBRN were 33.63 mg/100 g and 51.60 mg/100 g, respectively. Germination significantly increased the GABA content in brown rice noodles, likely due to the activation of endogenous glutamate decarboxylase in brown rice catalyzing glutamic acid conversion to GABA [9,10]. Numerous studies have demonstrated that germination increases GABA content [6,38].

Polyphenols are among the most abundant bioactive compounds in brown rice, existing in both free and bound forms [39]. Table 3 shows that the free phenolic acid contents in BRN and GBRN were 129.13 µg/g and 157.16 µg/g, respectively, while the bound phenolic acid contents were 384.81 µg/g and 412.28 µg/g, respectively. This indicates that germination significantly increased the phenolic acid content in BRN. This increase may stem from the activation of key enzymes responsible for phenolic acid synthesis during germination. For instance, phenylalanine ammonia-lyase (PAL) contributes to the production of free and soluble phenolic acids, while cell wall peroxidase (CW-PRX) induces the formation of bound phenolic acids [40]. Similar results were obtained by Wu et al. [41], who found that the free and bound phenol content of sprouted brown rice increased significantly by 38.26% and 6.02%, respectively, after 18 h of sprouting.

Resistant starch cannot be digested in the small intestine and does not lead to an increase in the glycemic index. After germination treatment, the resistant starch content of brown rice noodles was reduced from 27.61% to 21.66%. A similar phenomenon was found in germinated chickpeas [42], millet, and buckwheat [43]. This decrease in resistant starch content can be attributed to several reasons: (1) during germination, starch was hydrolyzed, and its structure was disrupted, making it more susceptible to further hydrolysis by amylase [44]; (2) starch binding to proteins and fibers was looser, improving the accessibility of the enzyme; and (3) anti-nutritional factors, such as phytic acid, were degraded [45].

## 4. Conclusions

Developing brown rice products is beneficial for reducing grain loss and improving the nutritional quality of staple foods. However, noodles made from brown rice typically suffer from high cooking losses and poor eating quality. This study demonstrates that germination significantly reduces the cooking time, cooking loss, hardness, adhesiveness, chewiness, and bitterness of brown rice noodles. Additionally, it significantly increases the dietary fiber, γ-aminobutyric acid, and total phenolic content of the noodles while decreasing the resistant starch content. Overall, germination improves the textural properties of brown rice pasta and enhances its nutritional properties.

## Figures and Tables

**Table 1:** Effect of germination on the cooking quality and texture properties of brown rice noodles.

Samples	BRN	GBRN
Cooking times (s)	709.67 ± 5.51 *	667.33 ± 4.62
Cooking loss (%)	10.07 ± 0.68 *	8.30 ± 0.30
Breaking rate (%)	0.00 ± 0.00	0.00 ± 0.00
Hardness (g)	493.71 ± 11.20 *	438.15 ± 8.20
Adhesiveness (g·s)	1.25 ± 0.09 *	0.82 ± 0.04
Chewiness (g)	624.88 ± 13.90 *	473.46 ± 6.72

All data are expressed as mean ± standard deviations. “*” indicates that the samples are significantly different (*p* < 0.05). BRN: brown rice noodles, GBRN: germinated brown rice noodles.

**Table 2:** The effect of germination on the color of brown rice noodles.

Samples	L*	a*	b*	WI
Uncooked				
BRN	48.36 ± 0.72	1.59 ± 0.03	8.41 ± 0.34	47.65 ± 0.68
GBRN	48.43 ± 0.33	2.29 ± 0.06 *	10.43 ± 0.28 *	47.33 ± 0.38
Cooked				
BRN	69.52 ± 0.40 *	0.26 ± 0.02	10.67 ± 0.12 *	67.71 ± 0.35 *
GBRN	66.23 ± 0.16	0.70 ± 0.05 *	9.66 ± 0.31	64.87 ± 0.17

All data are expressed as mean ± standard deviations. “*” indicates that the samples are significantly different (*p* < 0.05). BRN: brown rice noodles, GBRN: germinated brown rice noodles.

**Table 3:** Effect of germination on the nutritional composition of brown rice noodles.

Samples	BRN	GBRN
Total starch (%)	81.79 ± 0.37 *	75.38 ± 0.63
Dietary fiber (%)	3.56 ± 0.13	6.15 ± 0.01 *
Lipids (%)	0.65 ± 0.00	0.73 ± 0.00 *
Proteins	6.90 ± 0.22	7.01 ± 0.16
GABA (mg/100 g)	33.63 ± 0.93	51.60 ± 0.54 *
Free phenolic acid (µg/g)	129.13 ± 0.97	157.16 ± 5.41 *
Bound phenolic acid (µg/g)	384.81 ± 10.68	412.28 ± 6.06 *
Resistant starch (%)	27.61 ± 0.55 *	21.66 ± 1.14

“*” indicates that the samples are significantly different (*p* < 0.05). BRN: brown rice noodles, GBRN: germinated brown rice noodles.

## Data Availability

The original contributions presented in the study are included in the article, further inquiries can be directed to the corresponding author.
